# Root Canals

**Published:** 1892-07

**Authors:** 


ARTICLE IV.

### ROOT CANALS.

If any dentist will go to the box which contains the teeth that he has extracted, and will make a careful study of their contours, he will at once see that there is a wide difference in the conformation of the crowns, and that the pulp chambers present as great a diversity in shape and size. He may also discover another fact, and that is that the entrance to the root canals is from all points of the compass, and presents all the modifications from a great round hole to the narrowest slit imaginable, while the canals themselves will be found ramifying in almost all directions. And yet, many seem to act upon the impression that all that is necessary to prepare a root for filling, is with a bur or drill to sink a shaft straight down into the tooth almost anywhere, and in some way it will find the root canal.

Of course, these remarks are mainly appilcable to attempts made in bicuspids and molars, but to some extent it is true of the anterior teeth. It is very easy in attempting to drill out the canal of an incisor, or in enlarging it for the insertion of a pivot, to break through somewhere at the side. It is not always possible to determine by external appearances in just what direction a root extends.

There may be a deflection just at the cervical portion, and the crown may be set at an angle to the axis. Sometimes, too, the root is not straight, but at a point between the crown and apex there is a curvature. Nor can this always be determined by probing with a flexible broach. Extreme care is, therefore, often necessary, in any attempt at enlargement of the canals, even in front teeth.

But if this be the case with single roots, what shall be said of irregular shaped bicuspids and molars? Sometimes in the latter the crown is exceedingly short, and the pulp chamber shallow, so that opening or enlargement of the latter with a bur is done at the peril of breaking into the septum between the roots. This, of course, is usually fatal to any attempt at their proper treatment and filling. Every effort to reach the canals is attended with the liability to enter the opening to the pericementum and alveolus, with the consequent influx of blood and serum, and the probability of the formation of a new point of infection.

The palatal root of a superior molar usually approaches in shape that of an incisor, and the canal is almost as readily found and entered. But occasionally it seems to be at one cornua of the pulp, and again nearer the other, and it requires considerable patient search before it can be exactly located. An engine bur is a very poor instrument to look for it with, because it gives no intimation of the fact when it is found, while it is qui e likely to go just where it is not wanted.

The posterior buccal root is yet more difficult to locate, and when found is hard to penetrate, while not infrequently it is absolutely impossible to thrust the finest broach for any distance into the anterior buccal root, or to enlarge it without extreme danger of opening through it at the sides. Sometimes the opening to the posterior root seems on a line with that of the palatal, and again it is the anterior one. Occasionally they all seem to diverge from near the same point, and unfortunately the contour of the crown gives little indication of what is the exact condition.

In lower molars, the posterior canal is usually easily located, but it is hard to determine its shape. The root may be wide and flat, with a thin, constricted portion in the center, and there may be an opening into each thickened border, while the central portion may be almost or entirely closed. Only the most careful examination will determine what is the exact condition.

The pulp canal of the anterior root of lower molars is the most aggravating of all. If, as is frequently the case, the cavity of decay is upon the posterior border of the crown, it is almost impossible, without cutting nearly through the tooth, to find the narrow slit that is too often all there is of it. The root, too, is frequently curved and very thin, while what of opening there is seems to exist at the very anterior edge of the crown. It is usually quite impossible to clean out such a root, and dependence can only be placed upon sloughing of the contents, the thorough asepticising of the canal by absorption of the medicament, and the final filling of it as well as one can, with a thin, plastic material. This method, of course, means treatment for a greater or less period of time.

Inferior bicuspids usually have but a single canal to fill, although this may be a wide but thin slit. There is commonly little difficulty in locating it, although the first bicuspid is more apt to give trouble than the second.

Superior bicuspids are more easily reached than the molars. The first sometimes has two canal openings, that may be readily entered, while in other cases there seems to be but one. The pulp chamber, too, is at times irregular in shape, the canals tortuous and with constricted portions. Not infrequently such a narrowing will be found about three-fourths of the way up, having passed which there seems to be a kind of enlargement between that point and the apex, which it is extremely difficult thoroughly to clean out, or successfully to fill. Yet the continued success of the operation very largely depends upon perfect work at this point.

Complete access to any root canal depends upon getting the mouth of it well open. If the entrance is at an angle to the axis of the root, there will be an overhanging edge of dentine, partially blocking the opening, and if the inclination points forward this ledge must be removed before any thorough work can be done. It is just at this point that the danger arises, especially in molars, for if the root be thin, there is danger of perforating it. It many lower molars the septum of bone is thick, the bifurcation extends well up to the crown, and but a very thin layer of dentine and cementum separates the pulp chamber from this bony septum between the roots.

If a bur be used to remove the dentine at such canal openings, there is great danger that it will penetrate through and the tooth be ruined. Even though the pericementum be not reached, if the cementum be injured serious consequences may ensue. It should be remembered that this is living tissue, and is in relation with the pericementum. It cannot be devitalized like the dentine, without the loss of the tooth. Irritation of the cementum corpuscles may extend to the investing membrane and thus induce a pericementitis that will make extraction necessary. There is little doubt that many teeth have thus been lost through injury to the cementum, when the exact cause could not be determined.

From all these considerations, then, we deduce the conclusion that a bur is a very dangerous thing to introduce into a pulp chamber. If it be necessary to enlarge the opening into a canal, the executor is the only safe instrument, for it is comparatively easy to know what progress has been made with it, and the advance is slower. The bur buries itself in its own debris, so that its exact position cannot be definitely determined, while the tremulousness of the handpiece is fatal to all delicacy of touch.

But with the greatest care in searching for root canals, and with the utmost pains in the attempt to enter them, there will still remain a percentage that must remain unfilled. They are so minute, perhaps, that the most delicate instruments cannot be introduced, or they are so tortuous that it is impossible to follow them to the apex. If sufficient time be given for the sloughing of their contents, and if antiseptics be allowed to remain until they shall have thoroughly penetrated them by absorption, they may, probably, safely be left to nature's care. But if one would know how best to obtain access to root canals, he must do what we urged at the commencement of this article—study well the forms of extracted teeth.—*Editorial Dent. Pract. and Advertiser.*

---

